# Impact of ^18^F-FET PET on Target Volume Definition and Tumor Progression of Recurrent High Grade Glioma Treated with Carbon-Ion Radiotherapy

**DOI:** 10.1038/s41598-018-25350-7

**Published:** 2018-05-08

**Authors:** Charlotte Debus, Maria Waltenberger, Ralf Floca, Ali Afshar-Oromieh, Nina Bougatf, Sebastian Adeberg, Sabine Heiland, Martin Bendszus, Wolfgang Wick, Stefan Rieken, Uwe Haberkorn, Jürgen Debus, Maximilian Knoll, Amir Abdollahi

**Affiliations:** 1 German Cancer Consortium (DKTK), Heidelberg, Germany; 2 Translational Radiation Oncology, National Center for Tumor Diseases (NCT), German Cancer Research Center (DKFZ), Heidelberg, Germany; 3 Division of Molecular and Translational Radiation Oncology, Heidelberg Institute of Radiation Oncology (HIRO), National Center for Radiation Research in Oncology (NCRO), Heidelberg, Germany; 4 Heidelberg Ion-Beam Therapy Center (HIT), Department of Radiation Oncology, Heidelberg University Hospital, Heidelberg, Germany; 5 Division of Medical Image Computing, German Cancer Research Center (DKFZ), Heidelberg, Germany; 6 Department of Nuclear Medicine, Heidelberg University Hospital, Heidelberg, Germany; 7 Department of Neuroradiology, Heidelberg University Hospital, Heidelberg, Germany; 8 Department of Neurology, Heidelberg University Hospital and Clinical Cooperation Unit Neurooncology, National Center for Tumor Diseases (NCT), German Cancer Research Center (DKFZ), Heidelberg, Germany; 9 Clinical Cooperation Unit Nuclear Medicine, German Cancer Research Center (DKFZ), Heidelberg, Germany

## Abstract

High-precision radiotherapy (HPR) of recurrent high grade glioma (HGG) requires accurate spatial allocation of these infiltrative tumors. We investigated the impact of ^18^F-FET PET on tumor delineation and progression of recurrent HGG after HPR with carbon ions. T_1_ contrast enhanced MRI and ^18^F-FET-PET scans of 26 HGG patients were fused with radiotherapy planning volumes. PET-positive (PET+) tumor volumes using different isocontours (I%) were systematically investigated and compared with MRI-derived gross tumor volumes (GTV). Standardized uptake ratios (SUR) were further correlated with GTV and tumor progression patterns. In grade IV glioma, SUR > 2.92 significantly correlated with poor median overall survival (6.5 vs 13.1 months, p = 0.00016). We found no reliable SUR cut-off criteria for definition of PET+ volumes. Overall conformity between PET and MRI-based contours was low, with maximum conformities between 0.42–0.51 at I40%. The maximum sensitivity and specificity for PET+ volumes outside of GTV predicting tumor progression were 0.16 (I40%) and 0.52 (I50%), respectively. In 75% of cases, FLAIR hyperintense area covered over 80% of PET+ volumes. ^18^F-FET-PET derived SUR has a prognostic impact in grade IV glioma. The value of substantial mismatches between MRI-based GTV and PET+ volumes to improve tumor delineation in radiotherapy awaits further validation in randomized prospective trials.

## Introduction

The prognosis of patients diagnosed with HGG remains poor, with median overall survival times of 10–15 months in grade IV glioma (glioblastoma multiforme, GBM) and 40–50 months in grade III glioma^1^. Local therapy failure poses a major obstacle in curative treatment of these patients^2,3^. High precision radiotherapy (HPR) holds the promise to escalate the dose in the tumor and improve local control while sparing normal tissue. Several ongoing trials are currently investigating the impact of HPR in treatment of recurrent high grade glioma (HGG)^4,5^. However, target delineation in HGG is challenging due to their infiltrative nature and heterogeneous tumor structure^6^. Current standard definition of the *gross tumor volume* (GTV) is primarily based on T_1_ weighted contrast-enhanced (T_1_ CE) magnetic resonance imaging (MRI)^7^. Potential extensions of the tumor and suspected microscopic infiltrations of the surrounding tissue are covered with an additional safety margin around the GTV, leading to the *clinical target volume* (CTV). More recent guidelines suggest the inclusion of volumes that are hyperintense on T_2_ fluid-attenuated inversion-recovery (FLAIR) MRI images, hence correlating with tumor-induced edema, into CTV^7^. However, to avoid toxicities such as radionecrosis in pre-irradiated normal tissue this region has to be maximally spared from high-dose volume in treatment of recurrent HGG. Therefore, the benefit of an improved local control via dose escalation with HPR could be compromised by narrow margins and inaccuracies in tumor delineation emphasizing the need for novel imaging modalities for precision medicine.

Biological imaging, e.g. assessment of tumor metabolism by amino-acid positron emission tomography (PET), may present a promising modality to assist in radiotherapy planning^8,9^. For tumor volume definition in recurrent HGG, L-[methyl-11C]methionine (^11^C-MET) PET and O-(2-[18F]fluoroethyl)-l-tyrosine (^18^F-FET) were studied among others^10–13^. ^18^F-FET PET is increasingly used in clinical routine^13–19^. However, selection of appropriate segmentation technique for identification of the PET active volumes is still controversially debated. So far, a consensus strategy for PET based tumor delineation is not established^20^. Manual segmentation of the PET active area is subject to inter- and intra-rater variations, based on the applied level window and experience of the contouring physician. Semi-automatic techniques, like region growing or thresholding, depend on the definition of seed points and threshold values that vary between users and institutions. A method often utilized in cases of ^18^F-FDG PET is the application of a fixed threshold on the *standardized uptake value* (SUV)^12^. Yet, uptake of ^18^F-FET in malignant tissue is much lower compared to ^18^F-FDG, and varies greatly between patients^21^. Using percentage thresholds on the maximum SUV, so-called isocontours, can normalize inter-patient differences in uptake to an extent, but the optimal threshold value to be used is unclear and literature values vary between 40% and 90%^14^.

A popular new approach is the normalization of maximum SUV values (SUV_max_) to the background signal of healthy brain, and applying a threshold on this ratio, but again the threshold values differ between authors^11,19,22^. Despite different methodological approaches employed most studies reported substantial mismatches between MRI based standard radiotherapy (RT) target definitions and ^18^F-FET active volumes. However, the extent of these mismatches has not been well studied and there is no consensus on a segmentation strategy that provides the optimum conformity or additional value of ^18^F-FET PET in RT target delineation. The presented study aims to quantify the mismatches and overlaps between T_1_ CE MRI and ^18^F-FET PET depending on the PET contouring technique. Furthermore, it was investigated whether the mismatches between the two modalities can be explained by tumor volumes not visible in the treatment planning MRI (i.e. areas of tumor recurrence) and should thus be included in the target volume.

## Methods

All procedures performed in this were in accordance with the 1964 Helsinki declaration and its later amendments or comparable ethical standards. The study was approved by the institutional ethics committee (ethics committee of the University of Heidelberg, S-421/2015).

### Patients, treatment planning and MRI

Imaging and radiotherapy treatment planning data from 26 patients with recurrent high-grade glioma was investigated in this IRB approved retrospective study. For this retrospective trial no informed consent was required. Patient characteristics are listed in Table 1. Out of the 26 patients, 12 were diagnosed with recurrent grade III glioma (3 secondary malignancies from astrocytoma grade II) and 14 with recurrent grade IV (GBM), including one secondary malignancy from a grade II astrocytoma.

**Table 1:** Patient Characteristics.

**Feature**	**Grade III**	**Grade IV**
**Age**		
<50	4	6
50–59	5	2
60–69	2	5
≥70	1	1
**Gender**		
male	9	9
female	3	5
**KPS**		
≤80	1	3
>80	11	10
NA	0	1
**Dose**		
Median	33 GyE	34.5 GyE
Range	30–42 GyE	30–42 Gye
**Status**		
PD	6	13
SD	4	0
Lost to FU	2	1

Patients received ^18^F-FET PET scans and contrast enhanced MRI prior to radiotherapy, with no more than three weeks in between. All patients were treated at Heidelberg Ion-beam Therapy center (HIT) with carbon ions. Target structures GTV and CTV were delineated by the treating physician based on the T_1_ CE MRI and treatment planning CT. GTV included the contrast enhancing structures on T_1_ MR images and, if applicable, resection cavities from previous treatments. Tumors were irradiated with varying fraction schemes (10 to 14 fractions, median 11 fractions, of 3 GyE each (Gray Equivalent according to the local effect model, LEM 1))^23^.

### ^18^F-FET PET scans

^18^F-FET PET scans were acquired using a Siemens Biograph 6 PET/CT scanner. Median injected activity was 190 MBq, ranging from 130 to 235 MBq. In 16 patients, a dynamic acquisition over 40 min was performed, and endpoint static ^18^F-FET PET images were calculated by averaging the scans of the last 10 minutes. The endpoint static images featured a resolution of 1.33 mm × 1.33 mm × 3 mm, acquired over 80 slices. In 10 patients, only one-point static scans 30 min post-injection were conducted (resolution: 1.33 mm × 1.33 mm × 3 mm taken over 54 slices).

### Magnetic Resonance Imaging

Images were acquired in the routine clinical workup using a 3 Tesla MR system (Magnetom Verio/Trio TIM, Siemens Healthcare, Erlangen, Germany) with a 12-channel head matrix coil. Briefly, the protocol included an isotropic T_1_-weighted 3D MPRAGE images (isotropic voxel size: 1 mm³) both before and after administration of a 0.1 mmol/kg dose of gadoterate meglumine (DOTAREM, Guerbet, France) as well as axial FLAIR and axial T_2_-weighted images (slice thickness 5 mm).

### Image analysis and statistics

Image processing was done using the Medical Imaging Interaction ToolKit MITK (www.mitk.org)^24^. MITK is an open-source software framework for medical image procession and image analysis. Its toolkit nature allows for expansion with own implementations. MITK offers all analysis tools necessary for the envisioned analysis, including registration, integration of radiotherapy structure sets, segmentation and statistical volume assessment, in combination with volume and image visualization. We implemented missing software tools for evaluation of PET image data. For dedicated analysis of PET imaging data, a tool for calculation of the SUV in each voxel was developed. The plugin requires definition of the time interval between tracer injection and measurement, the amount of injected activity, the patient body weight and the tracer isotope. These values can be entered manually or read directly from the DICOM data, if corresponding tags are set. The SUV is calculated voxelwise for the entire image. In nuclear medicine, segmentation of regions with increased tracer uptake is often done using isocontours. These isocontours apply a threshold defined by a percentage of the maximum SUV in the lesion of interest. For example, an isocontour of 70% includes all image voxels with values ≥70% of the SUV_max_ (note: in the Siemens syngo system, the definition of isoncontours is inverse, so that an isocontour of 70% includes values upto SUV_max_ − 70% = 30%). A plugin for segmentation with isocontours was developed, which enables flexible definition of the percentage threshold.

For statistical analysis and plotting the open-source *R* software package was utilized (version 3.3.2, http://www.R-project.org). Kruskal-Wallis as well as two sided, unpaired Wilcoxon tests were performed including multiple testing corrections, Bonferroni-Holms/false discovery rate (FDR). Linear or monotonic relationship between two variables was investigated with Pearson- and Spearman correlation, respectively. Mantel-Cox log-rank test was used to compare differences in Kaplan-Meier survival curves (KM). Cox proportional Hazards model was used for uni/multivariate analyses (survival package, version 2.40-1, https://CRAN.R-project.org/package=survival). The significance level was set to $\,\alpha =0.05$, two-sided tests were applied and 95% confidence intervals are reported. Sensitivity analyses together with post-hoc power/sample size calculations and bootstrapping approaches are presented where appropriate.

### Segmentation

**Figure 1:**
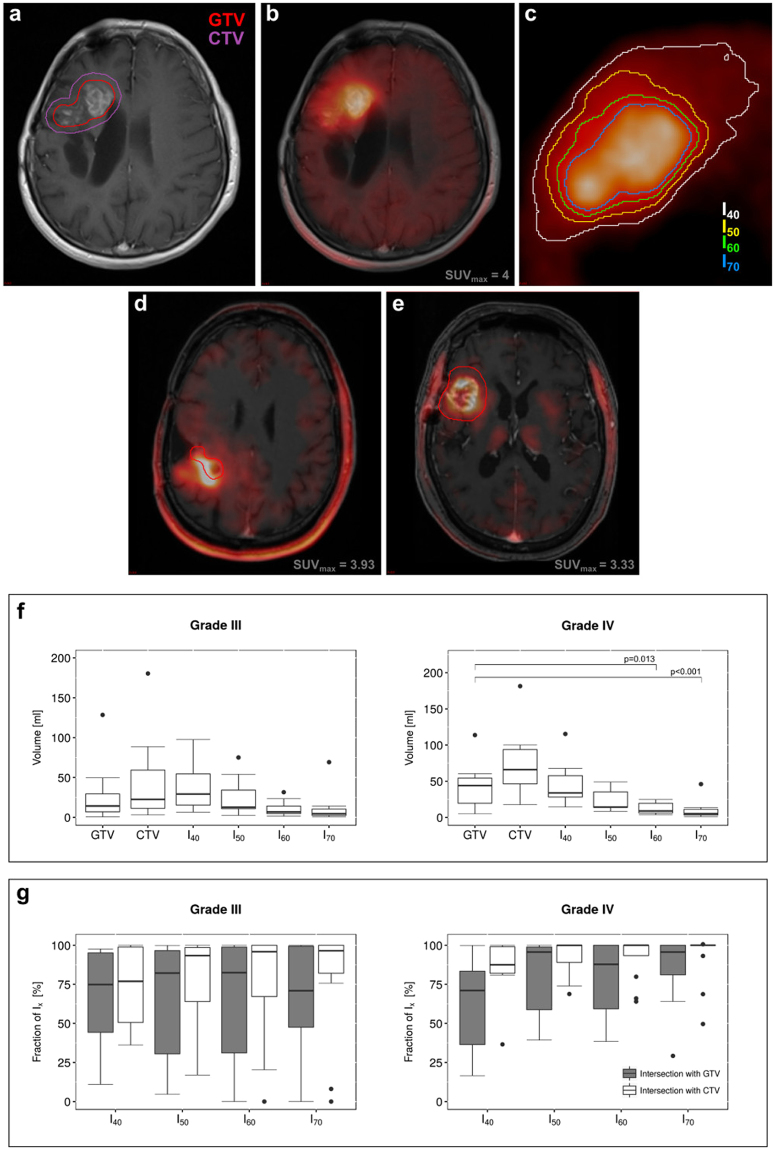
Amino acid PET with ^18^F-FET in radiotherapy treatment planning of recurrent high grade glioma. Current standard definition of radiotherapy *gross tumor volume* (GTV, red) and *clinical target volume* (CTV, purple) are based on T_1_ weighted contrast-enhanced (T_1_ CE) MRI (**a**). Amino-acid PET using ^18^F-FET is postulated to improve target delineation by assessment of tumor metabolism (**b**). However, defining an appropriate cut-off for PET-active tumor region is challenging, see e.g. different PET based tumor volumes based on selected thresholds (%) from the maximum intensity (isocontours I_x_, **c**). Comparative analysis of PET vs. MRI volumes (GTV, red) may result in substantial mismatched (**d**) or a relatively good overlap (**e**). Four different contouring techniques, using percentage threshold of the maximum standardized tracer uptake value (SUV_max_), i.e. 40% (white), 50% (yellow), 60% (green) and 70% (blue) isocontours were used for segmentation of ^18^F-FET active volumes (**c**). These volumes were then compared to T_1_ CE MRI based radiotherapy target volumes (GTV and CTV). Boxplots show the distribution of the individual volumes in grade III and IV recurrent glioma, respectively (**f**). Isocontours of 60% and 70% yielded significantly smaller volumes compared to GTV in grade IV glioma. Boxplots in (**g**) show the fraction of each isocontour volume that is included in either the GTV or CTV. Up to 50% of the PET derived isocontour volumes are not included in the GTV in grade IV glioma, whereas more than 75% of all isocontour volumes are included in the CTV volumes.

^18^F-FET PET images, treatment planning MRI images (T_1_ CE and T_2_ FLAIR), radiotherapy structure sets (GTV and CTV, illustrated in Fig. 1a) and follow-up MRI images (T_1_ CE) of each patient were exported from the clinical picture archiving system as DICOM data. Corresponding PET and MRI images were co-registered with MITK, using a multimodal rigid registration algorithm of the MatchPoint framework^25^, as shown in Fig. 1b. GTV and CTV from radiotherapy structure sets were mapped onto the co-registered images, as shown in Fig. 1a,c. The follow-up tumor volume GTV_FU_, based on the T_1_ CE follow-up MRI and the T_2_ FLAIR hyperintense volume CTV^FLAIR^ were contoured by radiation oncologists blinded to all other imaging sequences. The progressive tumor volume was defined as P = GTV\GTV_FU_. On basis of the ^18^F-FET PET images, four different isocontour thresholds were segmented as shown in Fig. 1c: 40%, 50%, 60% and 70%, referred to as I_40_, I_50_, I_60_ and I_70_. Hereinafter, the term ‘small isocontour’ refers to higher threshold values (i.e. 70%), as higher thresholds yield smaller volumes.

### Definition and quantitative comparison of tumor volumes

Volumes of GTV, CTV, CTV_FLAIR_ and the four different isocontours were measured using the MITK statistics plugin. The intersection between PET contours I_x_ and either GTV or CTV was calculated in order to investigate the coverage of ^18^F-FET active regions by these volumes:

1 $${V}^{GTV}={I}_{x}\cap GTV$$

2 $${V}^{CTV}={I}_{x}\cap CTV$$

Both volume fractions were normalized to I_x_, giving the percentage of coverage by either GTV or CTV.

**Figure 2:**
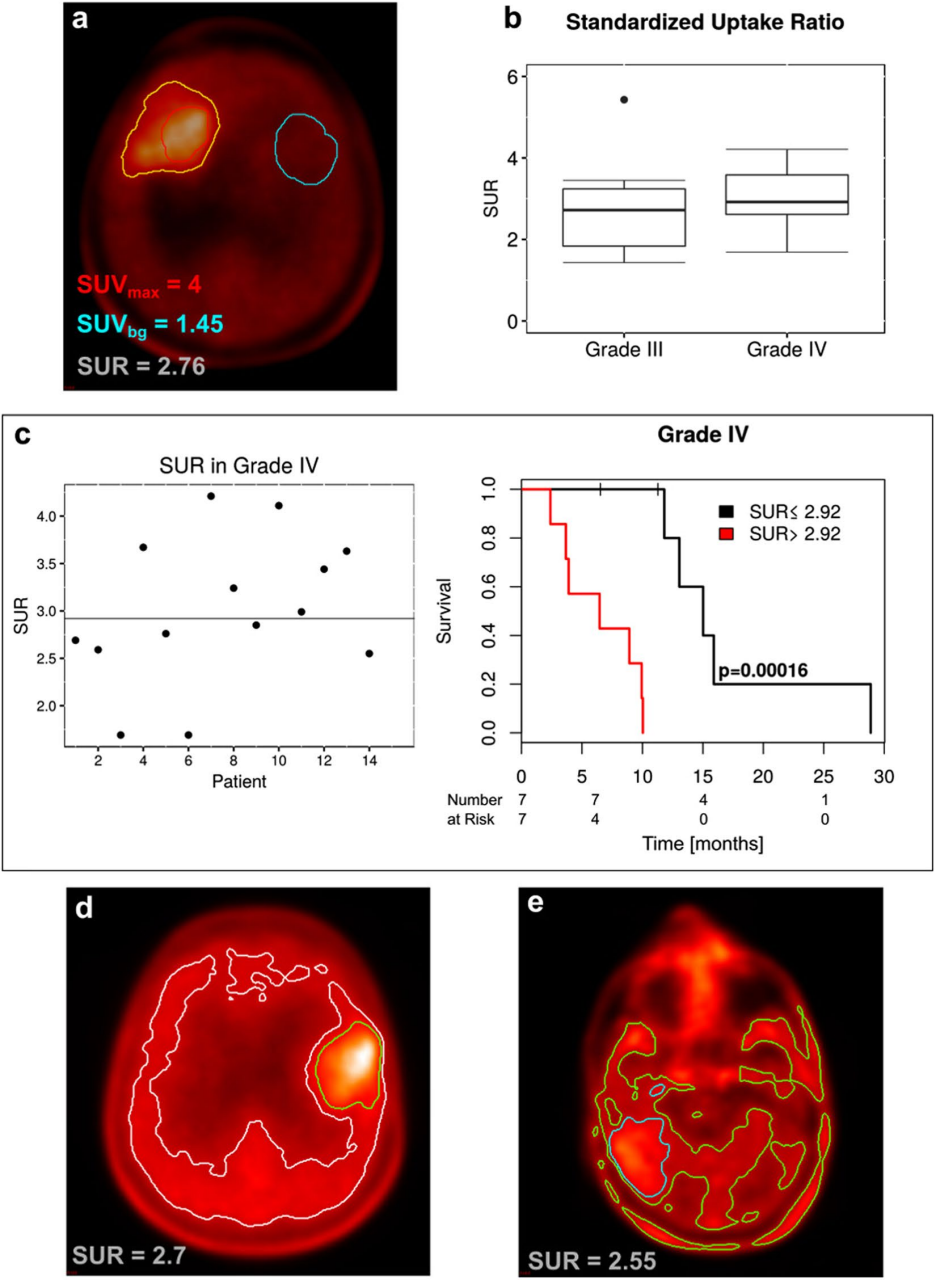
Prognostic relevance of ^18^F-FET PET based *standardized uptake ratio* (SUR). SUR is calculated by normalizing SUV_max_ in the tumor lesion to the average background SUV (SUV_bg_) within a region of similar size to GTV in the contralateral part of the brain, as shown (**a**). SUR was slightly but not significantly higher in grade IV glioma compared to grade III glioma (**b**). In grade IV tumors, the median value of SUR = 2.92 clearly separated patients into two prognostic subgroups as shown in KM-plot (**c**). This did not apply to grade III tumors (see supplements). For PET contouring, large interindividual heterogeneity of ^18^F-FET PET uptake was found limiting the use of a fixed threshold for all patients as illustrated in two patients (**d**,**e**). However, based on SUR, thresholds for the reasonable applicability of isocontours could be drawn as SUR > 2.73 for an isocontour of 40%, SUR > 2.55 for an isocontour of 50% and SUR > 1.62 for an isocontour of 60%. This was not possible using SUV_max_.

PET images were converted to SUV images using the aforementioned SUV calculation plugin. SUV_max_ was determined within the GTV. Mean background uptake (SUV_bg_) was derived by contouring a region of similar 2D size in a part of the brain contralateral to the tumor, as previously performed^13,26^ (shown exemplary in Fig. 2a). In patients with a tumor in one of the hemispheres, the background region was drawn at the centrum semiovale in the non-affected hemisphere. For midline tumors, the unaffected posterior or anterior half of the brain was used to draw this region. The *standardized uptake ratio* (SUR) was calculated by normalizing SUV_max_ to the background uptake:

3 $$SUR=\,\frac{SU{V}_{{\rm{\max }}}}{SU{V}_{{\rm{bg}}}}$$

The volume fraction A of the isocontour I_x_, which is not contained in GTV, was calculated as

4 $$A={I}_{x}\backslash GTV$$

Normalizing A to the GTV yields the percentage of GTV-increase by the PET active volume I_x_ ($A/{\rm{GTV}}\cdot 100 \%$), which influences the toxicity of the treatment.

For quantification of the overlap between isocontour I_x_ and GTV, the *conformity index* (*CI*) was defined as the intersection of both contours, normalized to their union:

5 $$CI=\frac{GTV\cap {I}_{x}}{GTV\cap {I}_{x}}$$

For each patient, the *best matching isocontour* was defined as the isocontour yielding the largest conformity index CI_max_. Corresponding threshold values of the SUR were calculated (${I}_{x}\cdot {\rm{SUR}}$), in order to relate the best matching isocontour to segmentations that are based on uptake-to-background thresholds.

Overlap between additional ^18^F-FET active volume A and progression of the tumor P was investigated by calculating the intersection J = A $\cap$ P. J was normalized to either A or P, giving the corresponding percentage value of coverage.

J/A labels the specificity of ^18^F-FET for detection of progression patterns, whereas the percentage of progression volume which is covered by the PET volume (J/P) is the sensitivity of ^18^F-FET for detection of tumor progression. Note: In conventional nomenclature, J/A is denoted as positive predictive value, as it is the ratio of true positives over all positives. Specificity in the classical meaning is the ratio between true negatives and the sum of false positives and true negatives. However, the amount of true negatives in this case is unknown, as it would be the volume of the entire brain which is not ^18^F-FET enhancing.

Finally, the intersection between PET contour I_x_ and CTV^FLAIR^ was calculated, in order to investigate the coverage of ^18^F-FET active regions by T_2_ FLAIR hyperintense volumes:

The fraction of I_x_ that is not included in CTV^FLAIR^ $({I}_{x}\backslash CT{V}^{FLAIR})$ was normalized to I_x_, giving the percentage of isocontour volume that is not covered by the T_2_ FLAIR hyperintense volume.

## Results

Median overall survival after this second course of radiotherapy was 27.5 months [4 to 60 months] in patients with grade III glioma and 9 months [2 to 28 months] in patients with grade IV glioma. 19 patients showed progression of the tumor after therapy, 6 in grade III glioma and 13 in grade IV glioma, leading to a median progression free survival of 7.5 months in grade III patients and 2 months in grade IV patients. Median follow up was 29.3 months.

The radiotherapy structure set (GTV, CTV) was fused with the MRI T_1_ CE and T_2_ FLAIR as well as with the gradually increasing thresholds for ^18^F-FET PET positive isocontours. Representative images of different isocontour volumes compared to the respective treatment planning GTV and CTV for grade III as well as IV glioma are presented in Fig. 1. The fraction of coverage of the respective isocontours by either GTV or CTV as percentage value is shown in Fig. 1d. The median GTV volume was larger in grade IV vs. grade III tumors (39.09 ml vs 10.78 ml, $p\,=\,0.0129$). The volumes of isocontours decreased with increasing threshold values reflecting the higher stringency for defining tumor positive regions by ^18^F-FET PET. I_60_ and I_70_ volumes were significantly smaller than GTV in grade IV tumors ($p\,=\,0.013$ and $p\,=\,0.00067$, respectively). Considering 10% increase in GTV by inclusion of the PET active volume a substantial change (${\rm{A}}/\text{GTV}\,\cdot 100 > 10 \%$), I_40_ added substantial volumes to the GTV in 2 cases (50%) for grade III glioma and in 7 cases (78%) for grade IV glioma. I_50_ added substantial volumes to the GTV in 4 (57%) (grade III glioma) and 5 (46%) (grade IV glioma) cases. I_60_ added substantial volumes in 4 (50%) (grade III) and 6 (46%) cases (grade IV). I_70_ added substantial volumes in 55% (6 cases, grade III) and 14% (2 cases, grade IV) of the patients.

### ^18^F-FET Uptake Ratio

Individualized tumor ^18^F-FET PET uptake ratios were measured by comparing the SUV_max_ with background (contralateral side, SUV_bg_, Fig. 2a). One patient presented with extremely low tracer uptake of SUR = 1.15 (SUV_max_ = 1.1) and was thus excluded from further analysis. The boxplot in Fig. 2b shows overall SUR for patients with grade III and grade IV glioma. SUR values in grade III glioma took a wide range of values (1 up to 5). In grade IV tumors, the range between 25^th^ and 75^th^ quantile was smaller ([1.61; 3.18] for grade III vs. [2.64; 3.55] for grade IV). There was a trend for higher SUR in grade IV vs. III glioma but it does not reach statistical significance (*p* = 0.262). In grade III tumors, SUR did not show any significant correlation with overall survival (Supplemental Fig. 1). In grade IV tumors, the median SUR clearly separated patients into a good prognosis (SUR > 2.92) with significant increase (~50%) of median survival (13 months) vs. poor (SUR < 2.92) prognosis group with a median survival of only 6 months (*p* = 0.00016, Fig. 2c). Accordingly, univariate analysis for correlation between SUR and survival yielded a hazard ratio of HR = 4.1 with 95% confidence intervals of [1.4–11.6] in grade IV glioma (*p* = 0.007). Significance of the results remained when including age and gender into a multivariate analysis; SUR: HR = 3.99, [1.4; 11.6], *p* = 0.013; Gender: HR = 0.67, [0.15; 3.1], *p* = 0.8; Age: HR = 1.02, [0.96–1.1], *p* = 0.49. Sensitivity analyses were conducted and parameter distributions obtained by boostrapping to allow further assessment of the results and to assure their validity (see Supplements).

Our data indicated that not every contouring technique could be applied in every patient. In patients with low tracer uptake, isocontours with lower thresholds, like I_40_ or I_50_, tend to yield unreasonably large volumes as they include scattered small segmentations from noisy background. Representative images of two different patients are illustrated in Fig. 2d,e. Due to low uptake, I_40_ failed in 12 patients (48%). I_50_ was not applicable in 7 patients (28%) and I_60_ in 4 patients (16%).

Careful examination of the individual contours in every patient and corresponding SUR of the lesion lead to thresholds for applicability of different isocontours: for I_40_: SUR ≥ 2.73, for I_50_: SUR ≥ 2.55, for I_60_: SUR ≥ 1.63. The thresholds were deduced as the lowest SUR value in the patient where application of the respective isocontour still yielded a reasonable segmentation.

### Conformity between MRI based GTV and PET isocontours

**Figure 3:**
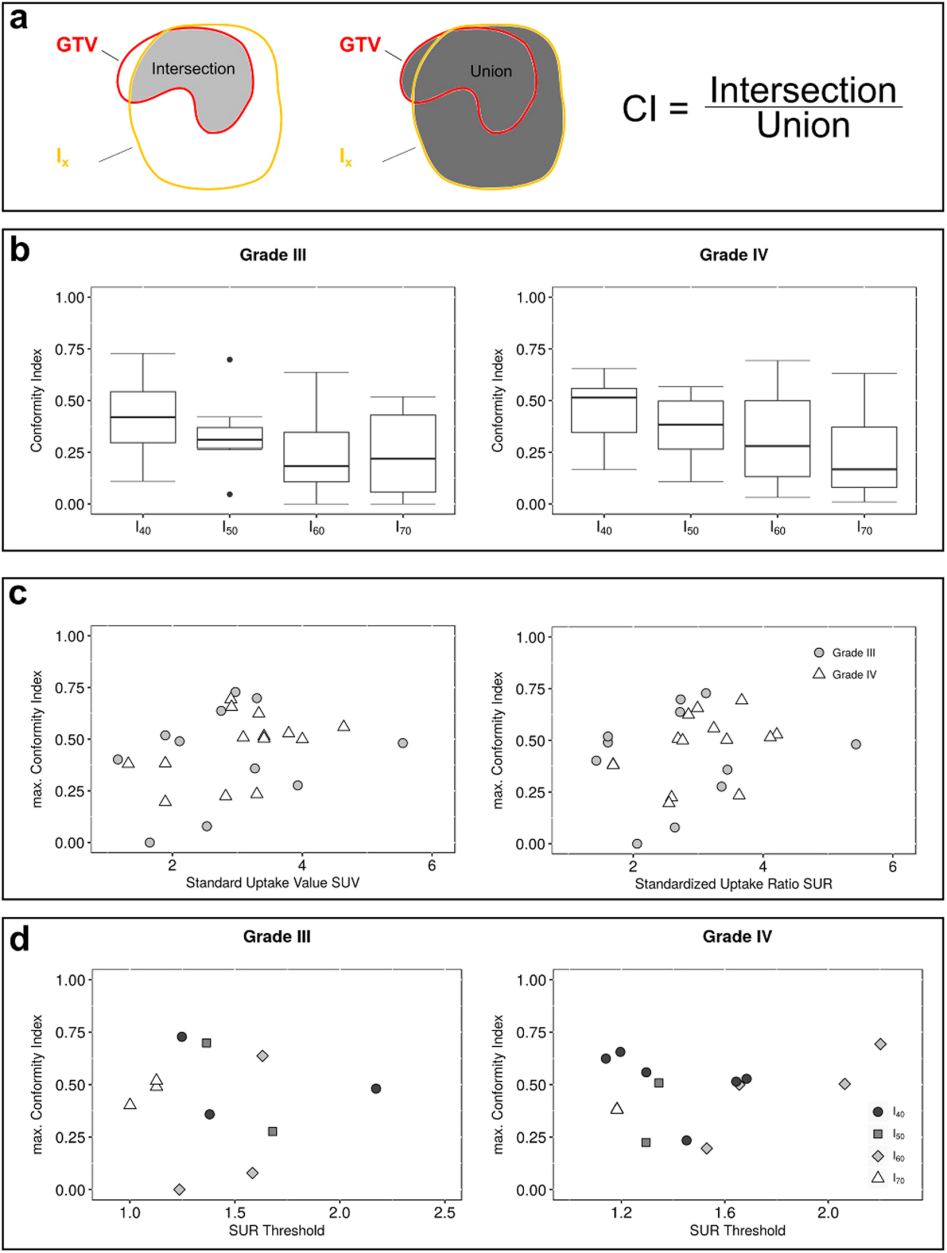
Quantification of overlap between different isocontour thresholds and MRI based GTV - the conformity index. The conformity index of each isocontour volume in a patient was calculated as intersection between isocontour and GTV normalized to their union, as schematically shown in (**a**). Boxplots in (**b**) show the distribution of conformity indices for every isocontour threshold. Higher thresholds (60%, 70%) yielded lower conformity, especially in grade IV glioma. The *best matching isocontour* was defined at the threshold yielding the highest conformity index with GTV. The corresponding highest conformity index is denoted as CI_max_. CI_max_ showed a non-significant trend towards correlation with SUV and SUR (**c**), especially in grade IV tumors, indicating that higher uptake tends to yield better conformity. Applying the *best matching isocontour* percentage threshold to the SUR of a patient yielded a corresponding SUR cutoff that the best matching isocontour would correspond to. For all given isocontour thresholds these cutoffs were distributed over a broad range of SUR values (**d**), and did not cluster into individual groups.

Our approach for quantification of overlap between the GTV and different PET isocontours (I_x_) and definition of the conformity index (CI) is schematically shown in Fig. 3a. Results of the conformity analysis are summarized in Fig. 3. The distribution of CI for the different isocontour thresholds are shown in 3b. Conformity decreases with higher isocontour thresholds, especially in grade IV tumors. Higher thresholds yield smaller volumes, and subsequently, higher isocontour thresholds result in smaller intersections with the GTV.

The maximum conformity index CI_max_, derived from the best matching isocontour, over SUV_max_ and SUR of the patient were calculated (Fig. 3c). In both grade III and IV tumors, a slight, but non-significant correlation between SUR or SUV_max_ of the lesion and maximum conformity index CI_max_, derived from the best matching isocontour, could be observed (Fig. 3c). The effect was strongest between SUR and CI_max_ in grade IV tumors, with a Pearson correlation of R = 0.36 and a Spearman correlation of R = 0.56 $(p\,=\,0.21)$. For all other correlation coefficients, refer to Supplementary Table 2.

Applying the percentage value of the best matching isocontour to the patients SUR value yields the corresponding SUR cutoff (or SUR threshold), a value often used for ^18^F-FET PET delineation in the literature. CI_max_ in dependence of the cutoff values of each patient are presented (Fig. 3d). The corresponding isocontours are labeled by different shapes. It can be observed that SUR cutoff values for individual isocontours do not cluster into separate groups, but are instead spread out over a wider range of SUR and CI_max_ values. Hence, our data illustrate the limitation of defining a fixed SUR threshold for ^18^F-FET PET contouring.

### Does PET-MRI discordance indicate regions at risk for tumor progression?

**Figure 4:**
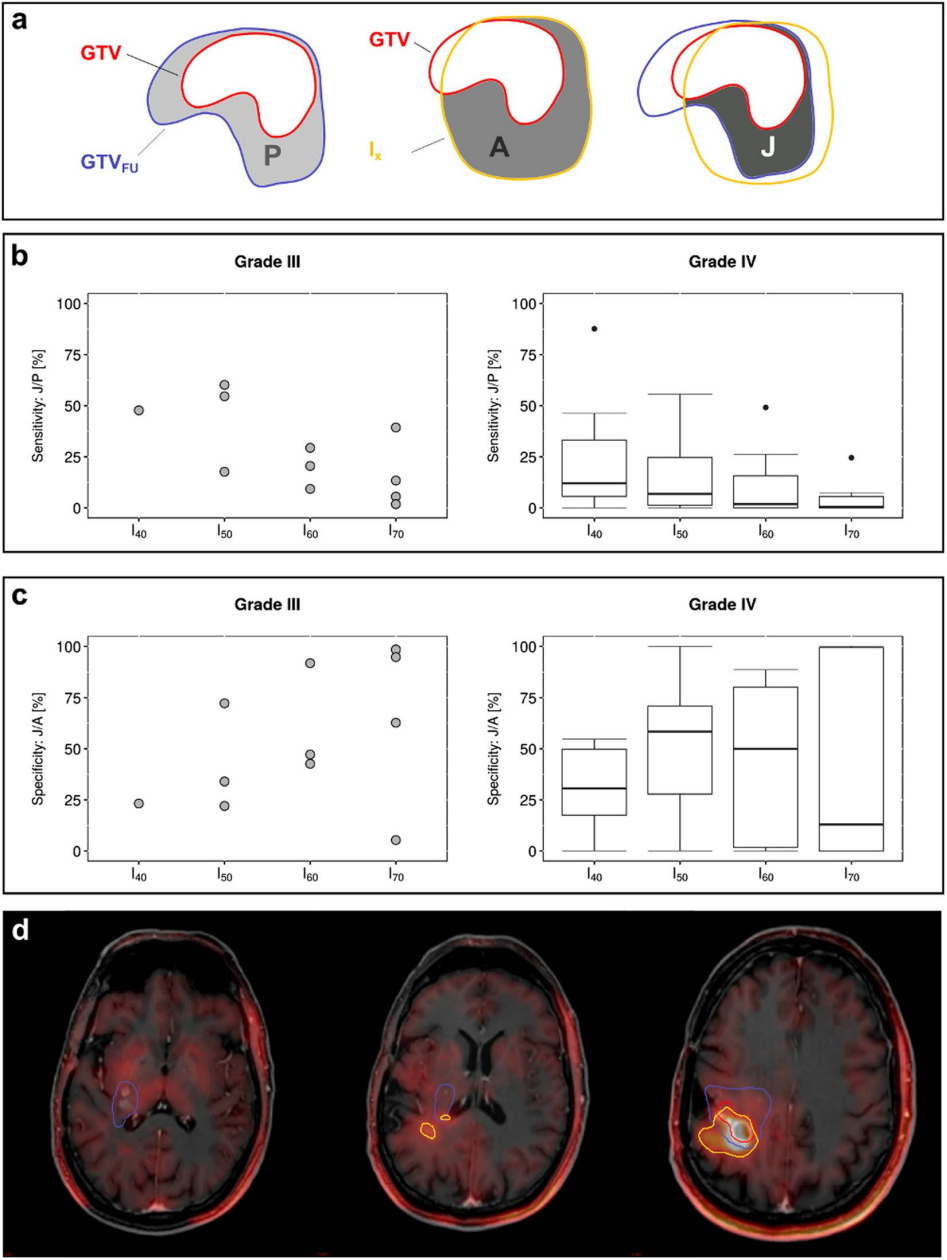
Detection of potential tumor progression by ^18^F-FET PET. The progressive tumor volume P was assessed by contouring the progressed tumor GTV_FU_ on the follow-up T_1_ CE MRI and calculating the difference to the original GTV: P = GTV\GTV_FU_. Of the 16 patients investigated, only n = 3 patients showed an in-field progression pattern. Therefore, the predominant failure pattern was “marginal” with 83% of patients (n = 12 patients) developing recurrences at the edges of the carbon ion field (CTV) underscoring the need for better delineation of the target volume. To evaluate, whether the PET isocontour volume A = I_x_\GTV outside of GTV could indicate parts of the progressed tumor volume, the intersection J between A and P was calculated with J = A\P, as schematically shown in (**a**). The sensitivity (**b**), percentage of progressive tumor volume that is covered by the additional PET active volume (J/P), and specificity (**c**), percentage of ^18^F-FET PET active volume that includes areas of tumor progression (J/A), of the additional ^18^F-FET active volume to detect regions at risk for tumor progress are shown. In grade III glioma, sensitivity decreases with increasing isocontour thresholds, whereas specificity increases with increasing isocontour thresholds. In grade IV glioma sensitivity decreased with increasing isocontour thresholds. Images in (**d**) show three axial slices in an example patient (grade IV) with low sensitivity and specificity, i.e. little overlap between ^18^F-FET PET (yellow line) and progress (blue line).

The identified mismatches between isocontours and GTV could indicate tumor regions at risk for progression. Therefore, the intersection (J) of the additional PET volume (A) with the tumor volume at progression (P) was evaluated. A denotes added PET volume outside GTV, i.e. PET-positive while MRI negative region at risk, at a given isocontour. J volume depicts added PET volume that corresponded with tumor growth at follow up. Together, specificity (J normalized to A) and sensitivity (J normalized to P) was identified for different PET isocontours (I_x_, Fig. 4).

From 19 patients who showed progression of the tumor after carbon ion therapy, complete follow-up imaging data was only available for 17, 5 with grade III glioma (from which one was excluded due to low tracer uptake, see above) and 12 with grade IV glioma. Interestingly, using the definition by Chan *et al*.^27^, of the 16 patients with progression investigated, only 3 showed an in-field progression (80–95% of progressive tumor within CTV) and 12 showed a marginal progression pattern (20–80% of progressive tumor within CTV). One case showed a distant progression pattenr (<20% of progressive tumor within CTV).

For grade III glioma, the individual values are shown as only 4 patients were found in this subgroup (Fig. 4b), and e.g. I_40_ was applicable in only one of them. Plots in Fig. 4b show a decreasing trend of sensitivity with higher isocontour thresholds. Whereas in grade III tumors, larger PET contours (I_40_ and I_50_) cover about half of the progressive tumor volume (J/P ≈ 50%), I_40_ covered only about 15% and I_70_ covered about 2% of the progressive tumor volume in grade IV tumors.

The percentage of additional PET volume that overlaps with the progressive tumor volume (J/A, specificity) appears to increase with higher isocontour thresholds in grade III glioma. This trend cannot be found in grade IV glioma, where I_60_ and I_70_ cover almost the entire spectrum, from 0% up to 90%. In both grade III and grade IV glioma, the range of values for J/A broadens with higher isocontour thresholds. Overall, coverage of the progressive tumor volumes is non-specific, and large parts of the additional PET active volumes seem to not correlate with tissue at risk for tumor progression.

In summary, these results indicate that only a small fraction of the progressive tumor volume is overlapping with the ^18^F-FET PET active tissue. Especially in grade IV tumors, ^18^F-FET demonstrates low sensitivity and low specificity to identify tumor tissue at risk for progression.

### Overlap of PET positive volume with T_2_ FLAIR based CTV

**Figure 5:**
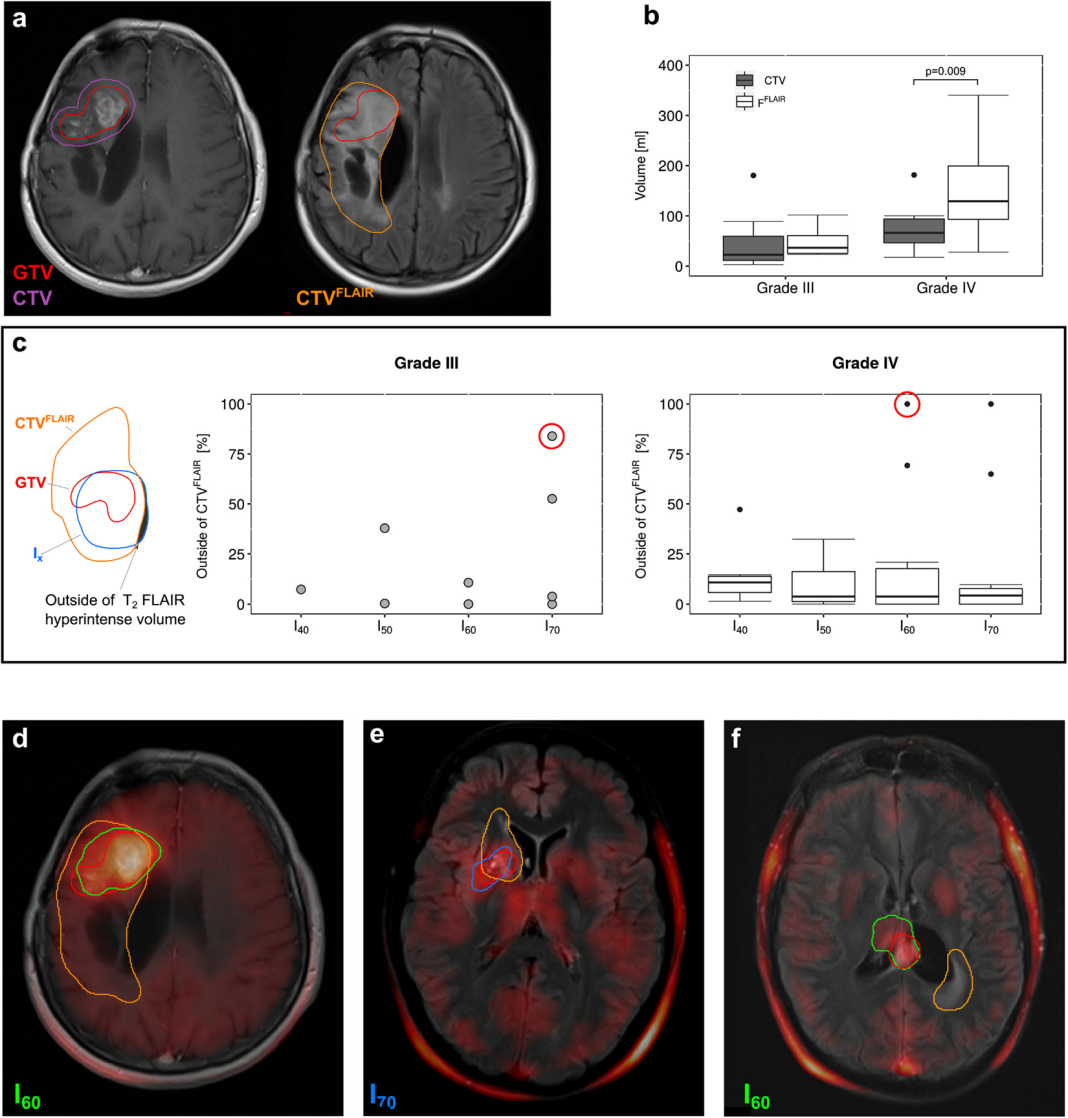
Coverage of ^18^F-FET PET isocontour volumes by T_2_ FLAIR hyperintense regions. Novel guidelines suggest inclusion of regions that are hyperintense on T_2_ FLAIR MRI (orange) into CTV^FLAIR^ (**a**). In grade IV glioma, this significantly increases the volume of CTV (**b**). The fraction of the ^18^F-FET PET based isocontour volumes that lies outside of the T_2_ FLAIR hyperintense volume, was calculated (**c**). With two exceptions for isocontours of 70% in grade III tumors and three exceptions for isocontours of either 60% or 70% in grade IV tumors, more than 80% of the isocontours were covered by the T_2_ FLAIR hyperintense volume (**c**). Representative patient with grade IV glioma where over 90% of the ^18^F-FET PET active area lies within CTV^FLAIR^ (**d**). Exemplary cases of two exceptional patients circled in red in the boxplots (**c**) with large fractions of the isocontour volumes (70% and 60%) laying outside the T_2_ FLAIR hyperintense volume are shown.

Novel consensus guidelines on radiotherapy target delineation suggest inclusion of T_2_ FLAIR hyperintense regions into CTV^7^. Our results indicate significant volume differences between current T_1_ CE based CTV and T_2_ FLAIR hyperintense volume CTV_FLAIR_ in grade IV tumors (*p* = 0.009, Fig. 5). In contrast, no significant difference between the two different CTV approaches was found in grade III glioma (*p* = 0.2). Furthermore, CTV^FLAIR^ was significantly larger in grade IV compared to grade III tumors (*p* = 0.026). Next, we studied the overlap between the ^18^F-FET regions and T_2_ FLAIR hyperintense volumes. 15 of the patients with tumor progression received T_2_ FLAIR scans for the treatment planning MRI. The percentage volume of the PET isocontours outside of CTV^FLAIR^ was quantified

6 $$\frac{{I}_{x}\backslash CT{V}^{FLAIR}}{{I}_{x}}$$

With a few exceptions, most of the PET volumes (~90%) are included in the T_2_ FLAIR hyperintense volumes, in both grade III and IV glioma (Fig. 5c). Two exemplary cases (one of each WHO grade) presenting with large mismatches between PET based contour and FLAIR volume are shown in Fig. 5d,e. The corresponding values are labeled by red circles in 5c.

Our results indicate that ^18^F-FET PET is well covered by CTV^FLAIR^ with limited added value for tumor delineation, especially when considering the low specificity of PET positive tumor volume to predict regions at risk for tumor progression.

## Discussion

Local therapy failure leading to tumor recurrence in proximity of the irradiated region is the major obstacle in curative treatment of HGG^28^. Current standard target volume definition in radiotherapy treatment planning of HGG is based predominantly on T_1_ weighted contrast enhanced MRI. ^18^F-FET PET may be a promising candidate to assist in radiotherapy tumor segmentation. Defining a meaningful ^18^F-FET PET activity cutoff to accurately identify vital tumor region versus surrounding normal tissue constitutes a major challenge. We utilized gradual intensity thresholds leading to different isocontours and systematically correlated the discordance between PET vs. MRI based tumor volumes with tumor growth pattern after carbon ion irradiation. Contour intersections were analyzed in terms of conformity, sensitivity and specificity of PET positive volumes to identify regions at risk for tumor progression. It was further studied whether a threshold for best matching SUV, SUR and isocontour could be defined.

We found no significant difference in median ^18^F-FET PET SUV/SUR between patients with grade III and grade IV glioma. This observation is in line with previously reported data (14, 15). However, reports also exist suggesting a correlation between ^18^F-FET SUR and glioma grade (16). One plausible explanation for the observed discrepancies was linked to possible differences in SUR outcome depending on the time point of PET scan after tracer injection (17). Therefore, dynamic ^18^F-FET PET scans might solve existing controversies.

We found that the median ^18^F-FET SUR of 2.92 robustly divided recurrent grade IV glioma into prognostic subgroups (HR: 4.1, *p* = 0.013). To our knowledge, this is the first evidence on the prognostic value of ^18^F-FET SUR for outcome of carbon ion irradiation. Interestingly, ~10% decrease in ^18^F-FET PET SUR after conventional radiochemotherapy was found to distinguish early responders in primary treatment of HGG^18^. Together with our observation, additional longitudinal ^18^F-FET SUR investigation in grade IV may be promising for discovery of novel prognosticators of radiotherapy response in this disease. Based on differences observed between grade III and grade IV glioma in our study, these tumor entities may be investigated separately in future ^18^F-FET PET trials.

We found no consensus SUV/SUR cut-off to reliably select ^18^F-FET positive isocontours to delineate tumor volume. Less stringent isocontours tend to yield better conformity with the MRI contrast-enhancing tumor for grade III glioma. In grade IV tumors, I_40_ represented the best matching isocontour in most cases. The increase in treatment volume as indicated by ^18^F-FET uptake varies greatly between different isocontour thresholds. Consequently, different segmentation techniques may greatly influence the size of target volume definition and augment toxicity of the irradiation.

Despite existing recommendations for ^18^F-FET PET delineation strategies, their impact on radiotherapy target volumes and outcome as well as congruence with other standard imaging modalities is still controversially debated^20,29,30^. Defining a fixed threshold for SUV was not feasible as ^18^F-FET uptake is relatively low (vs. e.g. FDG uptake) and varies greatly between patients^12^. For isocontours, different threshold values (40–90%) are in practice, but the optimal threshold value to be used is still unclear^14^. This also applies to recommended SUR based thresholds^11,19,22^. Our results indicate that fixed SUR thresholds are not suitable for delineation of ^18^F-FET PET in radiotherapy target definition generating variable conformity with the MRI based GTV. However, mismatches between MRI and ^18^F-FET PET can also result from tumor growth in the time interval between acquisition of the two modalities. Hence, multimodal imaging with combined PET-MRI scanners might help in further characterizing and quantifying mismatches and thus clarify the additional value of ^18^F-FET PET.

Previous studies incorporating ^18^F-FET PET active regions into the target volume definition reported mismatches between MRI based and amino acid PET derived contours^10,12,22,29,30^. It was therefore postulated that the PET positive regions not covered by current MRI based methods may indicate regions at risk for local tumor failure due to underdosing of radiotherapy. Our data does not support this hypothesis in recurrent HGG. Tumor growth pattern analysis revealed that ^18^F-FET PET identifies regions at risk for tumor progression after carbon ion therapy with low sensitivity and low specificity. Therefore, inclusion of ^18^F-FET PET active regions may result in coverage of a low fraction of regions at risk for tumor progression at the cost of substantially larger irradiated volumes. As novel RT target delineation guidelines recommend the inclusion of T_2_ FLAIR hyperintense regions into the treatment planning CTV^7^, we investigated the impact of such a contouring strategy on the coverage of ^18^F-FET PET active volumes. Interestingly, less than 10% of the PET active volume was found to be outside of the T_2_ FLAIR volume. Consequently, introduction of CTV^FLAIR^ in radiotherapy planning would lead to substantial coverage of ^18^F-FET active regions.

We investigated patients with recurrent glioma because no surgical resection of the tumor was performed prior to the second course of irradiation, and thus, correlation between pre- and post-therapy MRI images was not biased by spatial deformations. The extent to which our findings could be translated to treatment of primary glioma remains to be elucidated. While initial conclusions could be drawn from the 26 investigated patients, prospective randomized trials such as NOA10/ARO2013-1^31^ could provide definitive proof and validate the impact of our findings. The prevailing failure pattern (>90%) of conventional radiotherapy are “central or in-field” progressions, i.e., >80% of the recurrent tumor volume is localized in the high-dose radiation field (CTV^28^). In contrast, we found that only ~17% of failure were in the carbon ion field and the majority (~83%) of tumors showed a “marginal” progression pattern at the borders of carbon ion CTV. Therefore, identification of the tumor infiltration area will be of utmost importance for defining the target volume and the success of high precision irradiation with carbon ions. Moreover, different biological models underlying carbon ion dose distributions^32^ were not considered in the present study, which might impact tumor progression pattern analysis. Further studies including irradiation dose distribution should be conducted, which will become available within the MITK framework for implementations of biological particle therapy dose models. Our data clearly suggest the use of ^18^F-FET PET as prognosticator for carbon ion treatment of grade IV glioma. Combination of PET with other novel molecular based prognosticators^33,34^ may assist in stratifying patients benefiting most from carbon ion irradiation.

## Conclusion

The missing target hypothesis postulates that current MRI does not allow accurate detection of infiltrative growth pattern of high-grade glioma (HGG) limiting the efficacy of radiotherapy. Tumor metabolic switch leads to selective uptake of amino-acids such as ^18^F-FET and has fueled the hope to improve radiotherapy via a better delineation of HGG. We report high interindividual heterogeneity of tumor ^18^F-FET uptake limiting selection of a common reliable and robust threshold. Moreover, PET-positive volumes outside the current MRI-based tumor definition showed low sensitivity and specificity for identifying regions at risk for tumor progression. The prevailing patterns of radiotherapy failure are central or in-field recurrences. In our study, the predominant progression pattern was at the margins of the carbon ion irradiation field underscoring the urgent need for precise target definition for the success of this novel therapy modality. Patients with grade IV glioma showed significantly longer overall survival in cases of lower tumor ^18^F-FET uptake, demonstrating the prognostic value of amino acid PET.

## Electronic supplementary material

Supplementary Analysis
