# Supplementary material for: Impact of 18F-FET PET on Target Volume Definition and Tumor Progression of Recurrent High Grade Glioma Treated with Carbon-Ion Radiotherapy
Source: Sci Rep. 2018 May 8;8:7201. doi: 10.1038/s41598-018-25350-7 (PMC5940831; doi:10.1038/s41598-018-25350-7)
Supplement: Supplementary file 1 — Supplementary Analysis [file 41598_2018_25350_MOESM1_ESM.pdf]

Impact of ^{18}F-FET PET on Target Volume Definition and Tumor Progression of Recurrent High Grade Glioma Treated with Carbon-Ion Radiotherapy

Charlotte Debus[1-4,#,] , Maria Waltenberger[1-4], Ralf Floca[3,5], Ali Afshar-Oromieh[6], Nina Bougatf[3,4] , Sebastian Adeberg[3,4], Sabine Heiland[7], Martin Bendszus[7], Wolfgang Wick[1,8], Stefan Rieken[3,4], Uwe Haberkorn[6,9], Jürgen Debus[1-4], Maximilian Knoll[1-4] , and Amir Abdollahi[1-4,#]

[1]*German Cancer Consortium (DKTK), Heidelberg, Germany*
[2]*Translational Radiation Oncology, National Center for Tumor Diseases (NCT), German Cancer Research Center (DKFZ), Heidelberg, Germany*
[3]*Division of Molecular and Translational Radiation Oncology, Heidelberg Institute of Radiation Oncology (HIRO), National Center for Radiation Research in Oncology (NCRO), Germany*
[4]*Heidelberg Ion-Beam Therapy Center (HIT), Department of Radiation Oncology, Heidelberg University Hospital, Germany*
[5]*Software development for Integrated Diagnostics and Therapy, German Cancer Research Center DKFZ), Heidelberg, Germany*
[6]*Department of Nuclear Medicine, Heidelberg University Hospital, Germany*
[7]*Department of Neuroradiology, Heidelberg University Hospital, Germany*
[8]*Department of Neurology, Heidelberg University Hospital and Clinical Cooperation Unit Neurooncology, National Center for Tumor Diseases (NCT), German Cancer Research Center (DKFZ), Heidelberg, Germany,*
[9]*Clinical Cooperation Unit Nuclear Medicine, German Cancer Research Center DKFZ), Heidelberg, Germany*

Supplements

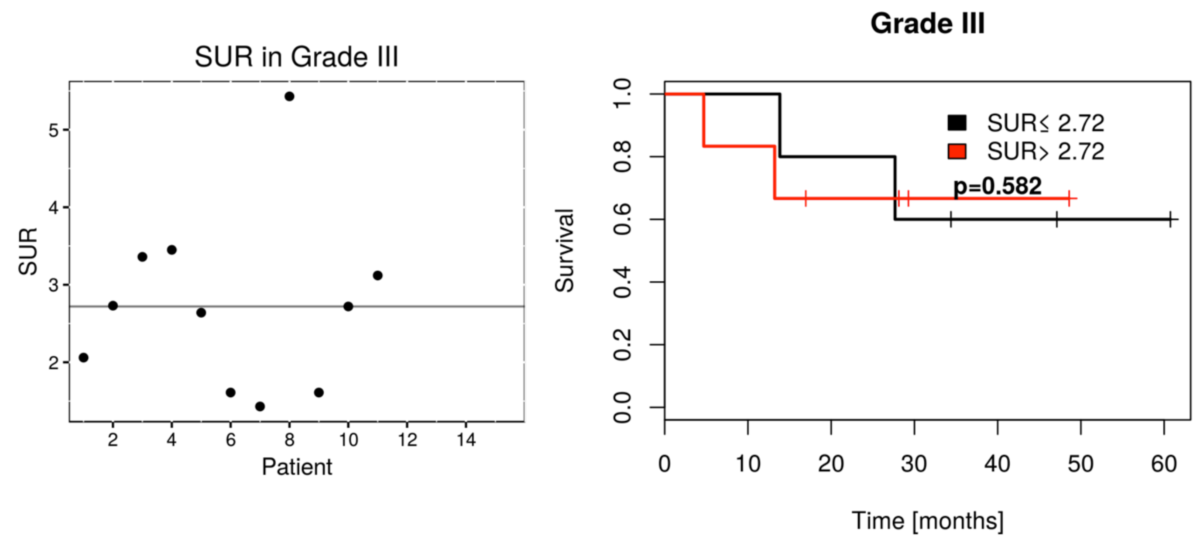

Supplementary figure 1: Relation between SUR and Survival in grade III glioma. The median value of SUR=2.72 separated two groups. However, the differences between the two groups were statistically not significant (log-rank, p=0.582). Overall, the median survival was not reached in both groups and the number of patient was limited for a decisive conclusion.

Correlation with		All	Grade III	Grade IV
SUV_{max}	Pearson	0.31	0.22	0.43
	Spearman	0.31	0.2	0.42
SUR	Pearson	0.21	0.11	0.35
	Spearman	0.29	0.05	0.56

Supplementary table 1: Correlation coefficients R for relationship between SUV_{max}/SUR with the maximum conformity index CI_{max} (c.f. Fig. 3c)

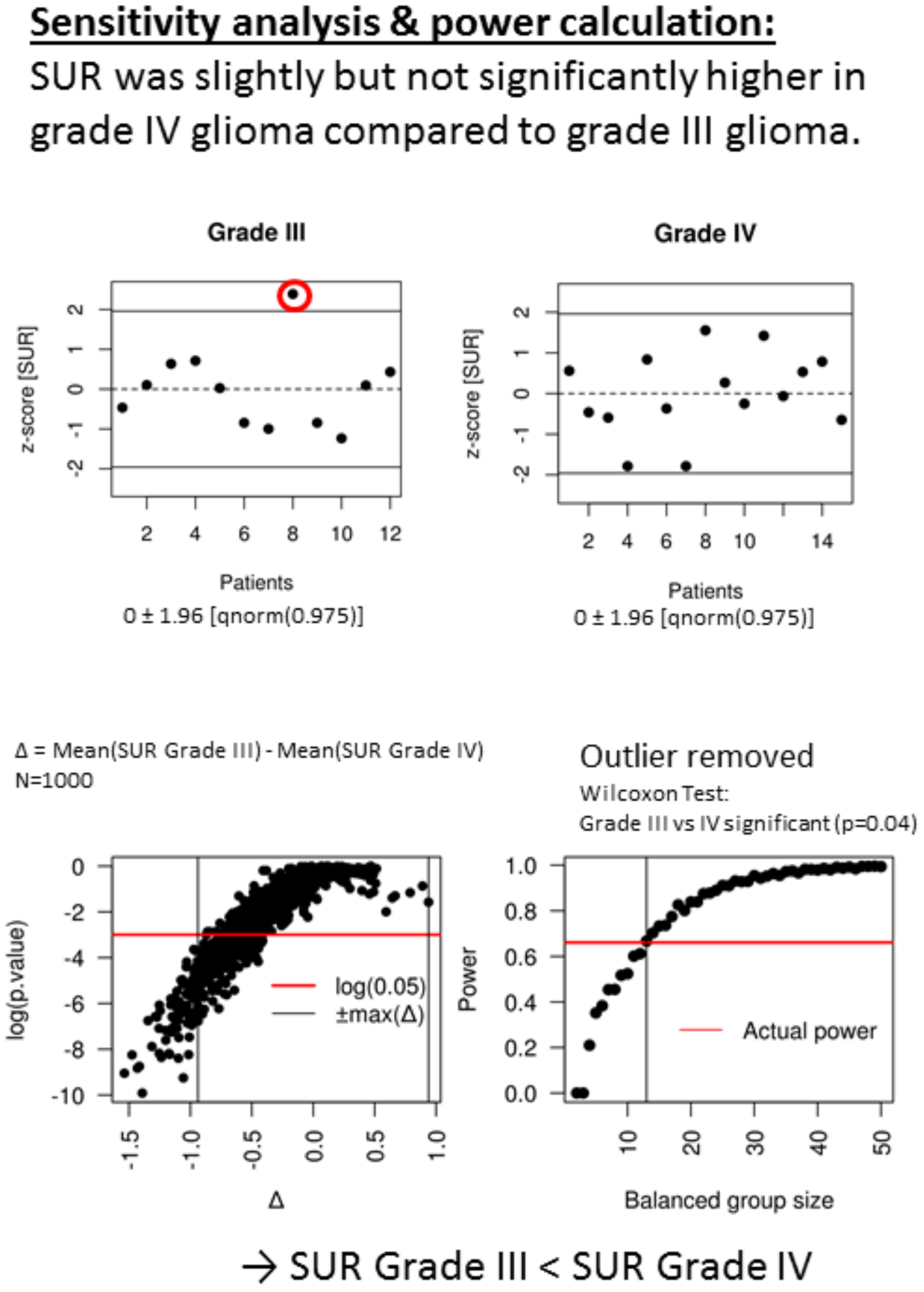

Supplementary figure 2: Sensitivity analysis of the SUR difference between grade III and IV tumors. Potential outliers in SUR measurements were identified by examination of standardized values (z-transformation). Calculation of effect differences between groups and the corresponding Wilcoxon p-value (two-sided) for 1000 bootstrapped datasets (including the outlier). Post-ho power calculation using the observed measurements (outlier removed) for a balance group design.

Power calculation

In grade IV tumors, the median value of SUR=2.92 clearly separated patients into two prognostic subgroups as shown in KM-plot.

Only one covariate (SUR)

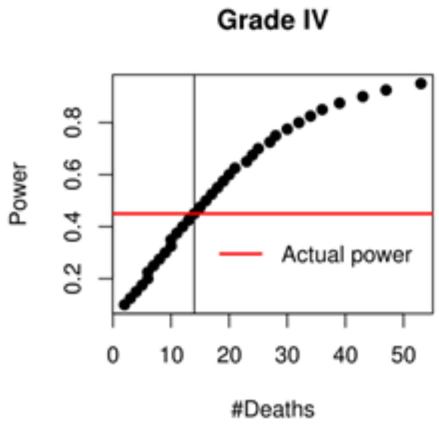

Bootstrap
CoxPH Model (N=1000)

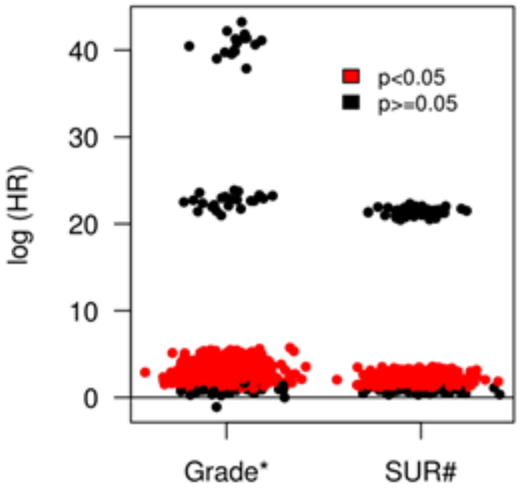

Multivariate: Grade + SUR

	Hazard Ratio	95% CI	p-value
Grade*	9.36	2.85-30.76	<0.001
SUR#	3.22	1.16-8.95	0.025

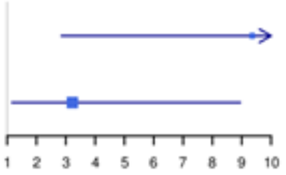

Two covariates: Grade + SUR

Required number of deaths: 34

* Grade IV vs III
\# high vs low

Supplementary figure 3: Multivariate survival analysis (tumor grade and SUR) for the observed and bootstrapped data as well as power and sample size calculation using estimates derived from the presented data
